# The segregation of different submicroscopic imbalances underlying the clinical variability associated with a familial karyotypically balanced translocation

**DOI:** 10.1186/s13039-015-0205-9

**Published:** 2015-12-30

**Authors:** Ana Carolina S. Fonseca, Adriano Bonaldi, Simone A. S. Fonseca, Paulo A. Otto, Fernando Kok, Mads Bak, Niels Tommerup, Angela M. Vianna-Morgante

**Affiliations:** Department of Genetics and Evolutionary Biology, Institute of Biosciences, University of São Paulo, Rua do Matão, 277, 05508-090 São Paulo, SP Brazil; Wilhelm Johannsen Centre for Functional Genome Research, Department of Cellular and Molecular Medicine, University of Copenhagen, Copenhagen, Denmark; Department of Neurology, School of Medicine, University of Sao Paulo, Sao Paulo, Brazil

**Keywords:** Familial karyotypically balanced translocation, 5q23.2-23.3 duplication, *LMNB1* duplication, 2p14 deletion, *CEP68, RAB1A* and *ACTR2* deletion, 2p14p15 microdeletion syndrome

## Abstract

**Background:**

About 7 % of karyotypically balanced chromosomal rearrangements (BCRs) are associated with congenital anomalies due to gene or regulatory element disruption, and cryptic imbalances on rearranged chromosomes. Rare familial BCRs segregating with clinical features are a powerful source for the identifying of causative genes due to the presence of several affected carriers.

**Case presentation:**

We report on a karyotypically balanced translocation t(2;22)(p13;q12.2) associated with variable learning disabilities, and craniofacial and hand dysmorphisms, detected in six individuals in a three-generation family. Combined a-CGH, FISH and mate-pair sequencing revealed a ten-break complex rearrangement, also involving chromosome 5. As the consequence of the segregation of the derivative chromosomes der(2), der(5) and der(22), different imbalances were present in affected and clinically normal family members, thus contributing to the clinical variability. A 6.64 Mb duplication of a 5q23.2-23.3 segment was the imbalance common to all affected individuals. Although *LMNB1*, implicated in adult-onset autosomal dominant leukodystrophy (ADLD) when overexpressed, was among the 18 duplicated genes, none of the adult carriers manifested ADLD, and *LMNB1* overexpression was not detected in the two tested individuals, after qRT-PCR. The ectopic location of the extra copy of the *LMBN1* gene on chromosome 22 might have negatively impacted its expression. In addition, two individuals presenting with more severe learning disabilities carried a 1.42 Mb 2p14 microdeletion, with three genes (*CEP68, RAB1A* and *ACTR2*),which are candidates for the intellectual impairment observed in the previously described 2p14p15 microdeletion syndrome, mapping to the minimal overlapping deleted segment. A 5p15.1 deletion, encompassing 1.47 Mb, also detected in the family, did not segregate with the clinical phenotype.

**Conclusion:**

The disclosing of the complexity of an apparently simple two-break familial rearrangement illustrates the importance of reconstructing the precise structure of derivative chromosomes for establishing genotype-phenotype correlations.

**Electronic supplementary material:**

The online version of this article (doi:10.1186/s13039-015-0205-9) contains supplementary material, which is available to authorized users.

## Background

About 7 % of karyotypically balanced chromosomal rearrangements (BCR) are associated with congenital anomalies [1]. Truncation of dosage sensitive genes [2, 3] or regulatory genomic landscapes [4], and cryptic imbalances on the rearranged chromosomes [5] are often the underlying pathogenic mechanisms in disease-associated BCRs. Rare familial BCRs segregating with clinical features contribute to the identification of causative genes due to the presence of several affected carriers [6, 7]. We describe a three–generation Brazilian family with six individuals carrying a karyotypically balanced chromosomal translocation t(2;22)(p13;q12.2) associated with variable learning disability and craniofacial and hand dysmorphisms. By array-comparative genomic hybridization (a-CGH), fluorescent in situ hybridization (FISH), and mate-pair sequencing (MPS), we demonstrated that the apparently simple two-way balanced translocation was a more complex three-chromosome rearrangement, also involving chromosome 5, with three novel copy number variations: two microdeletions at 2p14 and 5p15, and a microduplication at 5q23.2-23.3. As a consequence of the segregation of the derivative chromosomes 2, 5 and 22, different imbalances were present in affected and clinically normal family members, thus contributing to the clinical variability.

## Case presentation

**Fig. 1:**
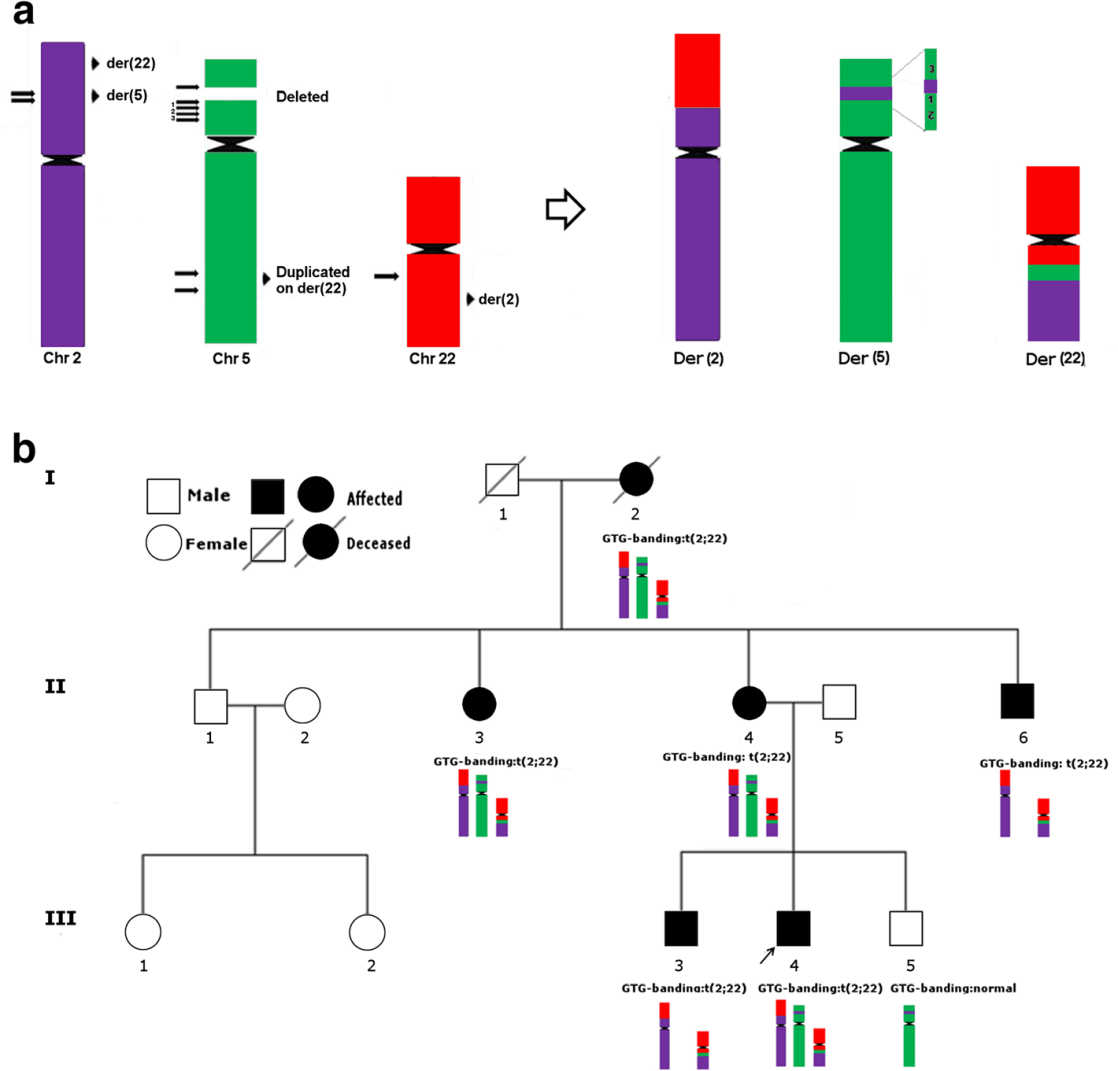
The structure of the derivative chromosomes der(2), der(5) and der(22), and their segregation. (**a**) In addition to the translocation of segments between chromosomes 2 and 22, a 1.42 Mb segment from 2p14 was found to be inserted into 5p15.1, where a 1.47 Mb deletion was detected; two other segments at 5p15.1, and one segment at 5p15.1-p14.3 was also rearranged on the der(5), one of them distal and two proximal to the 2p14 insertion; on chromosome 22 a duplicated segment of 6.64 Mb from 5q23.2-q23.3 was inserted into the breakpoint. Arrows point to breakpoints; localization of the resulting fragments is indicated at right. (**b**) The rearranged chromosomes – der(2), der(5) and der(22), were present in the proband (III-4), his mother (II-4), aunt (II-3) and grandmother (I-2), who therefore carried a 5p15 deletion and a 5q23.2q23.3 duplication; the proband’s affected brother (III-3) and uncle (II-6) inherited the der(2) and der(22), thus carrying a 2p14 deletion and a 5q23.2 duplication; the proband’s clinically normal brother (III-5) inherited only the der(5), and had a 2p14 duplication and a 5p15 deletion

The proband (III-4; Fig. 1), the second child of unrelated parents, was delivered at term by cesarean section, with a weight of 2,640 g (5^th^ percentile). For the first five months, feeding difficulties with regurgitation were frequent. The child was able to sit up without support at eight months, and walked independently at 16 months. Speech development was delayed: he spoke only isolated words until the age of four years. At six, he was referred to our Genetic Counseling Service due to learning difficulties. His height (117 cm) and weight (22 kg) were within the normal range (50^th^ - 75^th^ percentiles). The patient presented with turricephaly, flattened facies, short forehead, synophrys, epicanthal folds, normal inner (28.4 mm) and outer (80.0 mm) intercanthal distances (both between the 50^th^ and 70^th^ percentiles), long eyelids, low nasal bridge, high-arched palate, and small and low set ears. The hand abnormalities included brachydactyly, cutaneous syndactyly and clinodactyly of the 5th finger bilaterally, and a single transverse palmar crease at left. The first interdigital distance was increased in both feet. At a second clinical evaluation at the age of 15, height was 164 cm (25^th^ percentile) and weight was 62.9 kg (75^th^ percentile). Learning difficulties persisted and he was referred for speech therapy.

The older brother of the proband (III-3) was delivered at term, also after cesarean section. Birth weight was 3,250 g (25^th^-50^th^ percentiles). He walked independently at 14 months. At eight years, he was referred to our Genetic Counseling Service due to severe learning difficulties; he could not read. His height was 135 cm (90^th^ percentile) and weight was 33 kg (90^th^ - 97^th^ percentile). He had similar craniofacial dysmorphisms as his brother: turricephaly, flattened facies, high forehead, synophrys, epicanthal folds, low nasal bridge, high-arched palate, and low set ears; his inner and outer intercanthal distances were 30 mm (50^th^ - 75^th^ percentiles) and 82 mm (75^th^ - 90^th^ percentiles), respectively. The hand abnormalities included brachydactyly, cutaneous syndactyly, and clinodactyly of the 5^th^ finger. At the age of 17 years, height was 174 cm (between the 25^th^ and 50^th^ percentiles) and weight was 71.2 kg (between the 50^th^ and 75^th^ percentiles). Severe learning difficulties persisted.

The mother of the boys (II-4) was evaluated at 32 years of age. Her height was 155 cm (10^th^ - 25^th^ percentiles) and her weight was 73 kg (90^th^ - 97^th^ percentiles). She presented with turricephaly, mid facial hypoplasia, low set ears, and bilateral brachydactyly and clinodactyly of the 5th finger. She had learning difficulties. A younger brother of the proband (III-5) was clinically normal.

The maternal grandmother I-2 (deceased from heart failure at the age of 77 years), an uncle (II-6) and an aunt of the proband (II-3), all carriers of the translocation, presented with similar dysmorphisms and variable degrees of learning disabilities; the uncle was more severely affected. These three patients were not personally examined but their clinical conditions were assessed through photos and familial anamnesis.

## Methods

### Chromosome analysis (GTG-banding and FISH)

GTG-banding analysis was performed on metaphases from cultured peripheral blood lymphocytes.

### Fluorescent in situ hybridization (FISH)

FISH was performed with BACs and PACs (Additional file 1: Tables S1 and Additional file 2: Table S2) selected on the University of California – Santa Cruz – Genome Browser (UCSC; http://genome.ucsc.edu; hg 19), as previously described [8].

### Array-comparative genomic hybridization (a-CGH)

a-CGH was performed using the Agilent Human Genome 105 K CGH Microarray (Agilent Technologies Inc., Santa Clara, CA, USA). The Agilent Human Genome 60 k CGH Microarray (Agilent Technologies Inc., Santa Clara, CA, USA), was used for patient III-3 and III-6. The microarray chip was scanned on an Agilent Microarray Scanner. Data were processed by Agilent Feature Extraction software 9.5 and analyzed with Agilent CGH Analytics 3.4 Software with the statistical algorithm ADM-2, and sensitivity threshold 6.7. At least three consecutive oligonucleotides had to have aberrant log2 ratios to be called by the software.

### Mate-pair sequencing (MPS) library preparation and data analysis

Mate-pair library was prepared for the genomic DNA of the proband (III-4) using the Nextera Mate Pair Sample Preparation kit (Illumina, San Diego, CA, USA). Mate-pair libraries were sequenced on HiSeq2000 (Illumina), as paired-end 100 bp reads (2 × 100 bp). After adapter trimming, reads passing Illumina Chastity filtering (>0.6) were aligned to the human reference genome (hg19), using Burrows-Wheeler Aligner (BWA) [9]. Sequences with more than two mismatches were excluded, together with duplicated sequences corresponding to PCR amplification. Reads not aligning uniquely were discarded from further analysis. Paired-reads aligning to different chromosomes, with unexpected strand orientation or increase/decrease in insert size, were extracted for further analysis. These “discordant” paired-reads were analysed by SVDetect [10] to predict structural variants (SV). The MPS results were visualized on the Integrative Genomics Viewer [11]. To identify sample-specific structural variants, the predicted SVs were compared with in-house mate-pair data sets, and SVs, which were not unique to the cases, were excluded. Only SVs supported by at least six independent pairs of reads were considered. The analysis focused on chromosomes 2, 5 and 22. Data from FISH and microarray analysis were used as guidelines to interpret the MPS data and to identify “missed” paired-reads necessary to delineate the derivative chromosomes.

### Quantitative reverse transcriptase polymerase chain reaction (qRT-PCR)

To evaluate the expression of *LMNB1, MARCH3* and *FBN2* genes mapping within the detected duplication at 5q23.2-23.3, qRT-PCR analysis was performed. Total RNA was extracted from peripheral blood leucocytes from the proband, his mother and two unrelated healthy controls (aged 25 to 30 year), using the NucleoSpin® RNA II kit (Macherey-Nagel, Düren, Germany). SuperScript III First-strand Synthesis (Invitrogen, California, USA) kit was used for preparing the cDNA. Primers for qRT-PCR were designed for one amplicon of the *LMNB1* (lamin B1), *MARCH3* (membrane-associated ring finger (C3HC4) 3, E3 ubiquitin protein ligase) and *FBN2* (fibrillin 2) cDNAs, using Primer 3 (Additional file 3: Table S3). The amplicons were normalized to the *ACTB* (actin, beta) gene. qRT-PCR reactions were carried out on an ABI 7500 real-time PCR machine, using the SYBR® green PCR master mix (Applied Biosystems). All samples were tested in triplicate. To calculate the relative and normalized levels of *LMNB1*, *MARCH3* and *FBN2* the data was analyzed using Excel (Microsoft Corporation, Redmond, WA) and an unpaired student's t test was performed for statistical analysis.

## Results

### Investigation of the translocation t(2;22) using a-CGH, FISH, and MPS

**Fig. 2:**
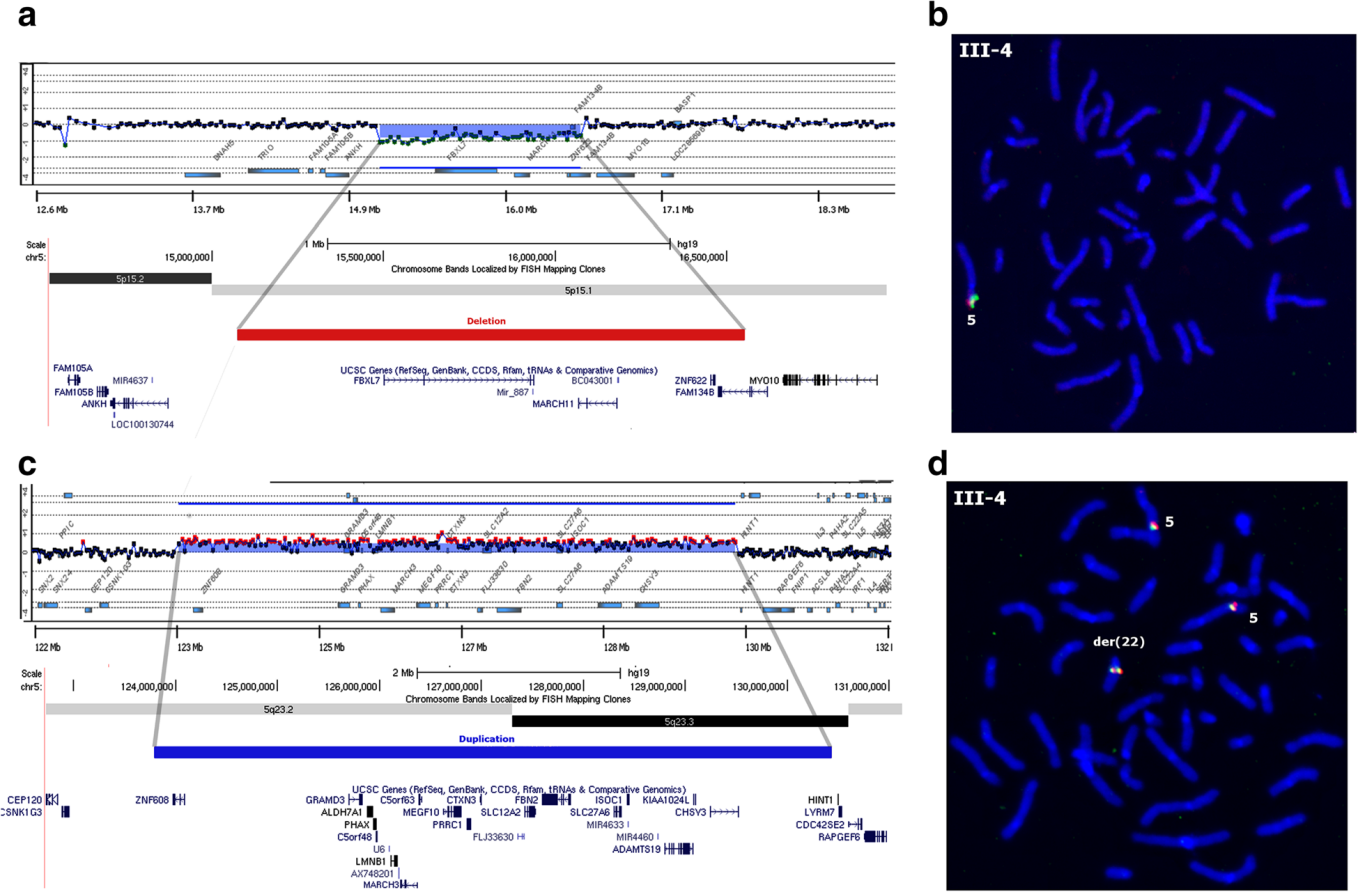
Chromosome 5 microdeletion and microduplication detected in the proband. (**a**) a-CGH (105 K, Agilent): Probes within a 1.45 Mb segment at 5p15.1 were deleted (chr5: 15,097,402-16,551,445; Human GRCh37 Assembly, hg19). The UCSC profile of the region depicts the deletion identified by a-CGH, which was extended to 1.47 Mb by MPS (chr5:15,073,606-16,552,845) (red), with the four genes mapping to this segment. (**b**) FISH probes RP1-137K24 (red signal) and RP1-167G20 (green signal) from the deleted segment, showed signals only on the normal chromosome 5. (**c**) a-CGH (105 K, Agilent): Probes within a 6.63 Mb segment on 5q23.2-23.3 were duplicated (chr5: 123,798,118-130,432,974). The UCSC profile depicts the duplication identified by a-CGH, which was extended to a 6.64 Mb interval by MPS (chr5:123,790,174-130,437,756) (blue), with the 18 genes mapping to this segment. (**d**) The additional segment of chromosome 5 was found to be inserted into the der(22) breakpoint region, by FISH, using the probes R11-48C14 (red signal) and RP1-236L2 (green signal) from the duplicated segment. This result was confirmed by MPS analysis (Table 1 and Additional file 7: Table S4)

**Fig. 3:**
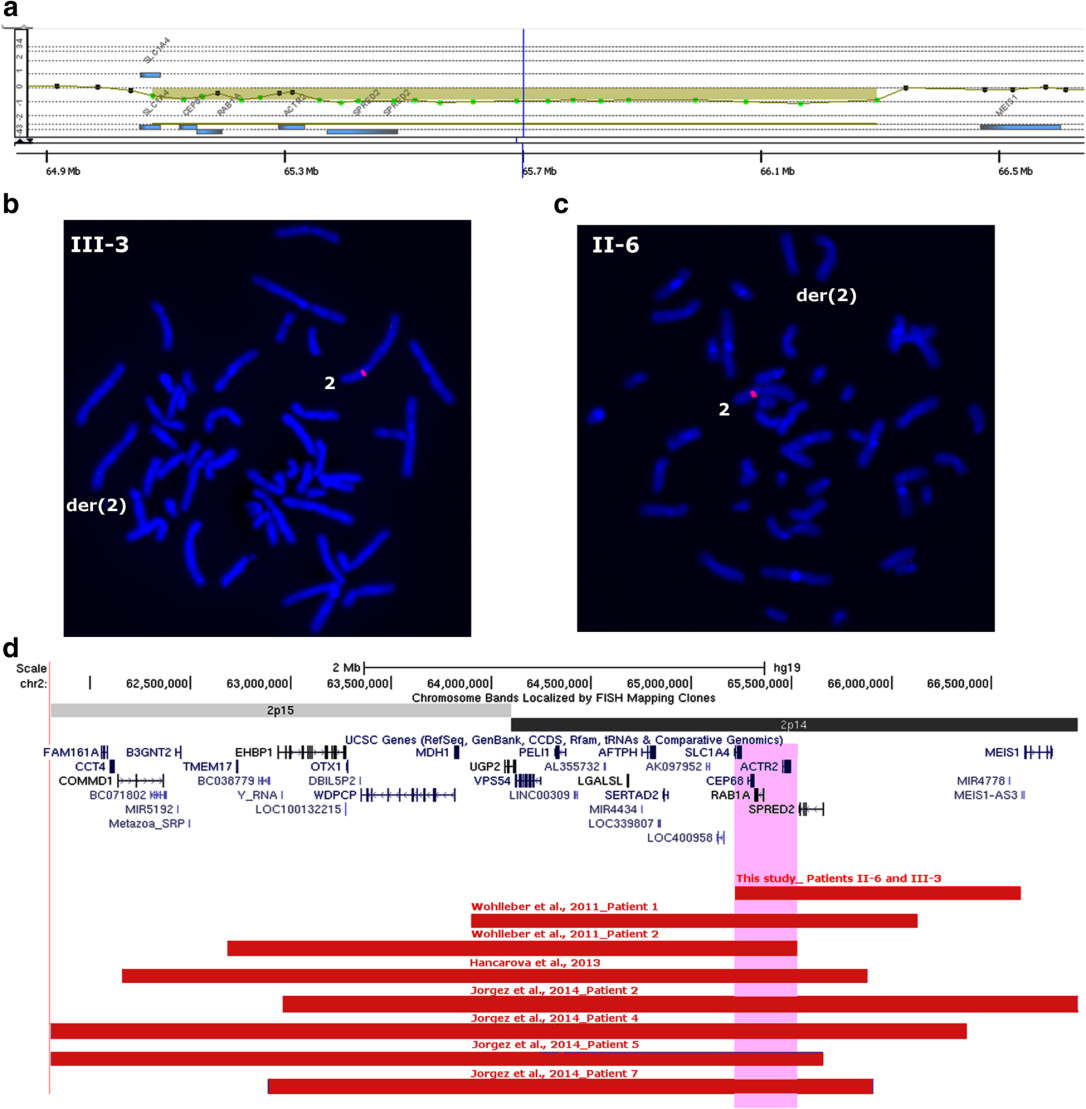
Microdeletion at 2p14 detected in two family members, and previously described overlapping deletions. (**a**) a-CGH (60 K, Agilent): Probes within a 1.24 Mb segment at 2p14 (chr2:65,237,764-66,484,321) were deleted in the proband’s affected brother (III-3). (**b**, **c**) FISH probe RP11-263L17 (red signal) from the deleted segment, hybridized to one chromosome 2 only, (**b**) confirming the deletion in II-3 and (**c**) showing that it was also present in III-6, the affected uncle of the proband. (**d**) The UCSC profile of the 2p15p14 region depicts the deletion identified by a-CGH, which was extended to a 1.42 Mb interval by MPS (chr2:66,646,777-65,220,481) (red). Seven overlapping microdeletions (red bars) associated with mild intellectual disability and minor dysmorphic features were previously reported [30–32]. The *CEP68, RAB1A* and *ACTR2* genes which maps to the minimal overlapping deletion interval are candidates for the intellectual impairment in the 2p14p15 microdeletion syndrome

After G-banding, a karyotypically balanced translocation t(2;22)(p13;q12.2) was detected in the proband (III-4), his older brother (III-3), mother (II-4), maternal grandmother (I-2), aunt (II-3) and uncle (II-6) (Fig. 1b). They shared similar clinical signs, although in variable degrees, II-6 and III-3 presenting with more severe learning disabilities. The clinically normal brother of the proband (III-5) had a normal karyotype. In the proband, a-CGH analysis revealed a 1.45 Mb deletion at 5p15.1 and a 6.63 Mb duplication at 5q23.2-23.3 (Fig. 2a-d). The deletion was confirmed by FISH (Fig. 2b), and the extra segment of chromosome 5 was found to be inserted into the der(22) breakpoint region (Fig. 2d). a-CGH and/or FISH detected the 5p15.1 deletion in three affected individuals [the mother (II-4), maternal grandmother (I-2) and aunt (II-3)], but also in the phenotypically normal brother (III-5) of the proband (Additional file 4: Figure S1). The insertion of 5q23.2-23.3 into the der(22), however, was detected only in the affected individuals (Additional file 5: Figure S2). Four and 18 genes are located within the deleted and duplicated segments of chromosome 5, respectively (Fig. 2a and c). In addition, a 1.24 Mb 2p14 deletion was identified by a-CGH in the proband’s affected brother (III-3; Figs. 1b and 3a-b). This deletion was also detected in the proband’s affected uncle (II-6; Figs. 1b and 3c). Five genes map to the deleted segment of chromosome 2 (Fig. 3d). In the phenotypically normal brother of the proband (III-5; Fig. 1b), this same 2p14 segment was duplicated, inserted into the short arm of the der(5) (Additional file 6: Figure S3). As expected, the other carriers of the der(5) – the proband (III-4), his grandmother (I-2), mother(II-4) and aunt (II-3), had the 2p14 segment inserted into der(5) as well (Additional file 6: Figure S3); they did not have any imbalance of chromosome 2, since they also carried the der(2). The affected brother (III-3) and uncle (II-6) of the proband both carried the deletion, since they inherited the normal chromosome 5 and not the der(5) from their respective mothers. No copy number variations were detected on chromosome 22.

The breakpoints (BPs) of chromosomes 2 and 22 were mapped by FISH (Table 1, Additional file 1: Table S1 and Additional file 2: Table S2). Due to the complexity of the a-CGH and FISH results, MPS was performed in the proband. This confirmed the seven BPs and the structural variations predicted by the combined karyotyping, FISH and a-CGH results (Table 1 and Additional file 7: Table S4), and further narrowed down the BPs to 1.5-5.7 kb segments. Based on the BPs delimited by MPS, the deletion and duplication on chromosome 5 were extended to 1.47 Mb and 6.64 Mb, respectively. As the 2p14 segment inserted into der(5) detected in the proband (III-4) corresponds to the 2p14 deleted segment in his affected brother (III-3) and uncle (II-6), and to the duplicated segment in the phenotypically normal brother (III-5), the 2p14 inserted/deleted/duplicated segment could be delimited to 1.42 Mb. In addition, three novel breakpoints on chromosome 5 were identified by MPS, proximal to the deletion at 5p15, and the resulting fragments were rearranged on the der(5), one of them distal and two proximal to the 2p14 insertion. The structure of the derivative chromosomes der(2), der(5) and der (22) based on a-CGH, FISH and MPS results are illustrated in Figs. 1a and Additional file 8: Figure S4. Three protein-coding genes (*SLC1A4:* solute carrier family 1 glutamate/neutral amino acid transporter, member 4*; FAM134B:* family with sequence similarity 134, member B; *TTC28,* tetratricopeptide repeat domain 28) and the non-coding RNA gene (*MEIS1-AS3:* MEIS1 antisense RNA 3) were disrupted by the rearrangement breakpoints (Table 1).

**Table 1:** Breakpoints (BPs) involved in the t(2;5;22), mapped by FISH, a-CGH and MPS, and truncated genes

Chr	BPs mapped by FISH		BPs mapped by a-CGH		BPs mapped by MPS		Band	Disrupted gene
	Position (hg19)	Interval (bp)	Position (hg19)	Interval (bp)	Position (hg19)	Interval (bp)		
chr2	65,209,004-65,265,044	56,040			65,218,054-65,220,481	2,427	2p14	*SLC1A4*
chr2	66,540,604-66,723,065	182,461			66,646,777-66,652,554	5,777	2p14	*MEIS1-AS3*
chr5			15,065,696-15,097,402	31,707	15,069,309-15,073,606	4,297	5p15.1	
chr5			16,551,445-16,590,384	38,940	16,552,845-16,557,142	4,297	5p15.1	*FAM134B*
chr5					18,178,446-18,182,480	4,034	5p15.1	
chr5					21,162,845-21,167,142	4,297	5p14.3	
chr5					25,131,744-25,133,892	2,148	5p14.1	
chr5			123,737,596-123,798,118	60,523	123,785,877-123,790,174	4,297	5q23.2	
chr5			130,432,974 – 130,476,355	43,382	130,437,756-130,442,053	4,297	5q23.3	
chr22	28,654,643-28,691,257	36,614			28,658,943-28,660,443	1,500	22q12.1	*TTC28*

### *LMNB1, MARCH3* and *FBN2* expression analysis

Although *LMNB1* is located in the 5q23.2-23.3 duplicated segment, transcript levels were normal in peripheral blood cells from the proband, and decreased in his mother, as demonstrated by qRT-PCR (Additional file 9: Figure S5). In contrast, increased expression of *MARCH3* and *FBN2* transcripts was detected in the proband and his mother, in accordance with their mapping within the 5q23.2-23.3 duplicated segment (Additional file 9: Figure S5). The decreased levels of *LMNB1* transcripts in the mother of the proband in comparison to her son might be due to an age-related effect [12].

## Discussion

We studied a karyotypically balanced translocation segregating in a family in association with learning disabilities of variable degrees and craniofacial and hand dysmorphisms. By combining a-CGH, FISH and MPS, the rearrangement, first identified by G-banding as a two-way balanced translocation, was found to be a ten-break rearrangement, resulting in six structural variations, which also involved chromosome 5 (Fig. 1a). This rearrangement presumptively originated in an ancestor of the proband’s grandmother, since she did not carry the der(5) with a deletion of the segment corresponding to the 5q23.2-23.3 fragment inserted into der(22); a meiotic exchange between the normal chromosome 5 and the original der(5) would have given rise to the detected der(5) with a normal long arm and an insertion of the 2p14 segment into the short arm (Additional file 8: Figure S4).

Despite the presence of three rearranged chromosomes – der(2), der(5) and der(22), only the der(2) and der(22) chromosomes segregated with clinical features (Fig. 1b). Common to all affected individuals were the 6.64 Mb duplication at 5q23.2-23.3, together with disruption of *SLC1A4* and *MEIS1-AS3*, on chromosome 2, and disruption of *TTC28,* on chromosome 22. The 1.47 Mb deletion at 5p15, which is a novel copy number variation (CNV) not reported in the Database of Genomic Variants (DGV), did not segregate with the clinical phenotype since the clinically normal brother of the proband (III-5) carried this deletion. He also carried another CNV not reported in DGV, a 1.42 Mb 2p14 duplication; his brother (III-3) and uncle (II-6) carried a deletion of this same fragment. Although this deletion might cause cognitive impairment, as discussed below, duplications of *CEP68, RAB1A, ACTR2* and *SPRED2*, located within the duplicated segment, have not been reported before. The microimbalances detected in the proband’s normal brother (III-5) highlight the importance of investigating unaffected individuals in the evaluation of the clinical impact of familial rearrangements.

There are some reports of larger 5q duplications, which include the present 6.64 Mb 5q23.2-23.3 duplicated segment [13]. However, these previously reported duplications varied in size, the smallest being 14 Mb, and differed in gene content, thus making phenotype-genotype correlations difficult to infer. Among the 18 genes encompassed by the 5q23.2-23.3 duplication in the present family, *LMNB1* is the only gene known to cause disease due to increased expression. *LMNB1* duplications cause adult-onset autosomal dominant leukodystrophy (ADLD; MIM 169500) [14]. Three other disease genes maps within the 6.64 Mb 5q23.2-23.3 duplicated segment: *FBN2 (*congenital contractural arachnodactyly*), ALDH7A (*pyridoxine-dependent form of epilepsy*)* and *MEGF10* (myopathy, areflexia, respiratory distress, and dysphagia). The disorders associated with these genes are caused by gain of function (*FBN2*) or loss of function mutations (*MEGF10* or *ALDH7A*), and it is unknown if overexpression of these genes is pathogenic. Also, 14 genes, which have not been implicated in diseases before, map within the duplication *(PHAX, MARCH3, SLC12A2, SLC27A6, ADAMTS19, CTXN3, CHSY3, ZNF608, GRAMD3, C5orf48, C5orf63, ISOC1, PRRC1, KIAA1024L*). Since this novel CNV was detected only in affected family members, duplication of one or more of these 14 genes likely contributes to the phenotype.

ADLD is a slowly progressive neurological disorder characterized by symmetrical widespread myelin loss in the central nervous system. The first signs of the disease, which includes cerebellar, pyramidal, and autonomic dysfunction, appear in the fourth and fifth decades of life [15]. None of the adults in the family described here, presently in the fourth and fifth decades of life [the proband's mother (aged 46 years), aunt (aged 53 years) and uncle (aged 45 years)], and the grandmother, deceased at the age of 77 years due to heart failure, showed symptoms of this disease. As determined by qRT-PCR, the *LMNB1* gene was not overexpressed in blood cells from the proband or his mother (Additional file 9: Figure S5). Increased *LMNB1* transcript level is detected not only in brain but also in blood from individuals affected by ADLD [16, 17]. On the other hand, in the proband and his mother, *FBN2* and *MARCH3* had a 2 to 3.8 fold increase in expression, demonstrating that the extra copy of both genes inserted into the der(22) were functional; these findings indicated that the normal and decreased expression of *LMNB1* in the proband and his mother, respectively, did not result from the silencing of the entire 5q23.2-23.3 segment on the der(22) due to “position effect”. Previously reported duplications including *LMNB1* in ADLD patients ranged in size from 128 kb to 478 kb; common to all these patients was a duplicated ~72 kb segment (chr5:126,102,443-126,174,517), encompassing the entire coding sequence of *LMNB1,* and the regions 9.8 kb upstream and 1.8 kb downstream the gene [17]. This interval was duplicated in the present family. Furthermore, a recently isolated *LMNB1* enhancer located 120 kb upstream of *LMNB1* [18] was also translocated to the der(22). However, the extra copy of *LMBN1* was located on the der(22) chromosome, while all previously reported *LMNB1* duplications in patients with ADLD were in tandem [14, 16, 17, 19, 20]. The disruption of *LMNB1* long range regulation or the interference of sequences on chromosome 22 (centromeric to *LMNB1*) or from the translocated chromosome 2 (telomeric to *LMNB1*) might have negatively impacted the expression of the *LMNB1* copy located on the der(22). Considering the position of the gene within the duplicated segment, disruption of *LMNB1* regulation would be due to the separation of an enhancer located more than 2.3 Mb upstream or 4.3 Mb downstream from *LMNB1.* The possibility also exists that other genes within the 5q23.2-q23.3 segment negatively affects *LMNB1* expression. Regardless of the mechanism interfering with *LMNB1* expression, this study illustrates the importance of evaluating the quantitative level of transcripts in carriers of duplications encompassing *LMNB1*, particularly considering that ADLD is a late-onset neurodegenerative disorder.

*SLC1A4* is also disrupted in all carriers of the der(2) chromosome in the present family. *SLC1A4* encodes a neutral small amino acid transporter, and is ubiquitously expressed [21], particularly in glial cells, and during brain development [22]. Recently, recessive mutations in *SLC1A4* were described in patients presenting with developmental delay, microcephaly and hypomyelination [23–25]. Most patients were homozygous for missense mutations (p.E256K, p.L315fs, and p.R457W) [23, 24], but one of them carried a missense (E256K) and a nonsense (Leu315Hisfs*42) mutations [24]. Mutated proteins were shown to abolish (p.R457W) or markedly reduce (p.E256K) L-serine transport [25], demonstrating that *SLC1A4* loss of function, rather than a gain of function mechanism, caused the clinical features. Heterozygous carriers of the missense and nonsense mutations in *SLC1A4* were clinically normal, arguing against a causative role of the *SLC1A4* truncation in the present family. The *TTC28* gene on chromosome 22 and the *MEIS1-AS3* non coding RNA on chromosome 2 were also disrupted by the breakpoints of the rearrangement. *TTC28* and *MEIS1-AS3* have not been associated with any known diseases and their phenotypic impact on the carriers of the der(2) and der(22) chromosomes remains unknown.

The t(2;5;22) could have also impacted the long range regulation of gene(s) located near the breakpoints. The breakpoint on chromosome 2 that disrupted *MEIS-S3* occurred approximately 10 kb from the *MEIS1* gene (Meis homeobox 1). SNPs within *MEIS1* were associated with the Restless Legs syndrome (RLS), a neurologic sleep/wake disorder [26, 27]. Although there is no evidence that *MEIS-S3* disruption affects *MEIS1* expression, the centromeric breakpoint on chromosome 2 occurred within the same topological domain as *MEISI* [28], and might have altered its long range regulation. Members of the reported family do not present RLS symptoms, but the role of *MESI1* in the development of the central nervous system [29] could implicate *MESI1* as candidate for the learning disabilities observed in the family.

Although the six translocation carriers presented with learning disabilities, these were more pronounced in the proband’s affected brother (III-3) and uncle (II-6), who were the only carriers of the 1.42 Mb chromosome 2 deletion. Seven microdeletions, all extending beyond the 1.42 Mb deletion described here, have been previously reported in association with intellectual disability and minor dysmorphic features, pointing to a 2p14p15 microdeletion syndrome [30–32] (Fig. 3d). The 1.42 Mb deletion detected in III-3 and II-6 is the smallest yet to be reported, encompassing *SLC1A4, CEP68* (centrosomal protein 68 kDa)*, RAB1A* (RAB1A, member the RAS oncogene family); *ACTR2* (ARP2 actin-related protein 2 homolog), and *SPRED2 (*Sprouty-related, EVH1 domain containing 2). *SLC1A4* was disrupted in all six affected family members, but, this variant is likely not pathogenic in the heterozygous state, as discussed above. *CEP68, RAB1A* and *ACTR2*, mapping to the minimal overlapping deleted segment appear as candidates contributing to intellectual impairment in the 2p14p15 microdeletion syndrome (Fig. 3d). *RAB1A* and *ACTR2* are involved in neuronal differentiation and regulation of axon guidance [30]. *CEP68* encodes a centrosomal protein; CEP proteins have an important role in neurogenesis in the developing human brain, as demonstrated by the clinical effect of homozygosity for recessive mutations in CEP genes, known to cause primary microcephaly [33]. However, while III-3 and II-6 had severe learning difficulties, the other seven patients with 2p14p15 microdeletions presented with intellectual disability [30–32]. This suggests that, in addition to the gene(s) encompassed by the deletion described here, other gene(s) contribute to the intellectual impairment in the 2p14p15 microdeletion syndrome. Most of the 2p14p15 microdeletion patients also present with genitourinary defects [31, 32] a feature associated with the loss of *OTX1* (orthodenticle homeobox1) at 2p15 [32]. The absence of genitourinary defects in III-3 and II-6, whose deletion does not include *OTX1*, is in line with the role of *OTX1* in the development of the genitourinary tract.

The challenge to establish genotype-phenotype correlations in carriers of chromosomal rearrangements extends beyond a particular DNA sequence to reach the genomic context [34]. Although several genes have been directly affected by the herein described t(2;5;22), this rearrangement might have impacted the genetic network by altering the physical relationship between chromatin domains, thus contributing to the clinical phenotype.

## Conclusions

The disclosing of the complexity and segregation of an apparently simple two-way rearrangement illustrates the importance of reconstructing the precise structure of derivative chromosomes for genotype-phenotype correlations. As the consequence of the segregation of the derivative chromosomes 2, 5 and 22, different imbalances were present in affected and clinically normal family members, thus contributing to the clinical variability. A 1.42 Mb 2p14 microdeletion, associated with more severe learning disabilities, pinpoints *CEP68, RAB1A* and/or *ACTR2* as candidate(s) for the intellectual impairment in the previously described 2p14p15 microdeletion syndrome. The absence of *LMNB1* overexpression, despite the presence of an additional copy of the gene, highlights the importance of genomic topology in disease, supporting the indication of evaluating genetic findings in the genomic context.

## Consent

This study was approved by the Ethic Committee for research involving human subjects at the Biosciences Institute, University of São Paulo (125/2011). Written informed consent was obtained from the patients or parents for publication of clinical data.

## Additional files

Additional file 1: Table S1. BAC clones used as probes for chromosome 2 breakpoint mapping in the proband (III-4). (XLSX 12 kb) Additional file 2: Table S2. BAC and PAC clones used as probes for chromosome 22 breakpoint mapping in the proband (III-4). (XLSX 11 kb) Additional file 3: Table S3. Primers used in qRT-PCR analysis of duplicated (*LMNB1, MARCH3* and *FBN2*) and reference (ACTB) genes. (XLSX 11 kb) Additional file 4: Figure S1. Segregation of the 5p15.1 deletion. FISH using probes RP1-137K24 (red signal) and RP1-167G20 (green signal), mapping to the deleted segment at 5p15.1, revealed signals only on the normal chromosome 5 on metaphase spreads of the mother (II-4), maternal grandmother (I-2), aunt (II-3), and in the phenotypically normal brother (III-5) of the proband. Both probes hybridized to both chromosomes 5 on metaphases of the affected brother (III-3) and uncle of the proband (II-6). These results demonstrated that the deletion at 5p15.1 did not segregate with the clinical phenotype in the family. (TIFF 2444 kb) Additional file 5: Figure S2. Segregation of the 5q23.2-q23.3 duplication. FISH using probes RP11-48C14 (red signal) and RP1-236L2 (green signal), mapping to the duplicated segment, revealed signals on both chromosomes and an addition signal on the derivative chromosome 22, on metaphases spreads of all affected individuals in the family. Hybridization signals were only observed on both chromosomes 5 in the proband’s normal brother (III-5). These results demonstrated that the duplication at 5q23.2-q23.3 segregated with the clinical phenotype in the family. (TIFF 2394 kb) Additional file 6: Figure S3. Segregation of the 2p14 insertion into the short arm of chromosome 5. FISH using probe RP11-263L17 (red signal), mapping at 2p14, hybridized to the normal chromosome 2 and to the derivative 5 on metaphase spreads of the proband (III-4), his grandmother (I-2), mother (II-4) and aunt (II-3). On metaphase spreads of the phenotypically normal brother of the proband (III-5), probe RP11-263 L17 hybridized to the normal chromosome 2 and to the derivative 5. Probe RP11-281O15, mapping at 5q35.3 (green signals), was used as control for patients I-2, II-4, III-4 and III-5. Probe RP11-325M10 mapping at 2q33.3 (green signal), was used as control for patient II-3. These results indicated that the 2p14 segment was inserted into the chromosome 5 short arm, the same chromosome harbouring the 5p15.1 deletion. The phenotypically normal brother of the proband (III-5), being a carrier of the der(5), had a 2p14 duplication. The proband’s affected brother (III-3) and uncle (II-6), who did not carry the der(5), had the corresponding deletion (Fig. 3). (TIFF 962 kb) Additional file 7: Table S4. Breakpoint junctions involved in the t(2;5;22) identified by mate-pair sequencing data. (XLSX 12 kb) Additional file 8: Figure S4. Mechanism of formation of the t(2;5;22) based on MPS data. **(A)** The rearrangement might have originated from ten occurring breaks (arrows): two on the short arm of chromosome 2, five breaks on the short arm and two on the long arm of chromosome 5, and one break on the long arm of chromosome 22. **(B**) The resulting fragments **(C)** rejoined randomly, a fragment from the short arm of chromosome 5 being lost. **(D)** This rearrangement presumptively originated in an ancestor of the proband’s grandmother, since she did not carry the der(5) with a deletion of the segment corresponding to the 5q23.2-23.3 fragment inserted into der(22). A meiotic exchange between the normal chromosome 5 and the original der(5) would give rise to the observed der(5) with a normal long arm and an insertion of the segment 2p14 into the short arm. **(E)** The derivative chromosomes 2, 5 and 22 detected in the family, and their normal homologues are depicted. (TIFF 3425 kb) Additional file 9: Figure S5.
*LMNB1*, *MARCH3* and *FBN2* expression determined by qRT-PCR. The diagram depicts the expression of *LMNB1, MARCH3* and *FBN2* in peripheral blood cells from the proband (III-4) and his mother (II-4), relative to healthy controls (*n* = 2). *LMNB1* transcript levels did not differ between the proband and controls, but were decreased in his mother, both carriers of the duplication at 5q23.2-23.3, encompassing the *LMNB1*. The decreased levels of *LMNB1* transcripts in the mother of the proband relative to her son might be due to an age-related effect [12]. On the other hand, increased transcript levels of *FBN2* and *MARCH3*, also mapping to the duplicated interval, were observed in both carriers. All samples were tested in triplicate (diagram represents mean values), and the *ACTB* expression was used to calculate the relative and normalized levels of *LMNB1, MARCH3* and *FBN2* transcripts. Error bars denote standard error of the mean (SEM). P values for fold change in expression are indicated (unpaired Student's t test). (TIFF 1845 kb)
