# Supplementary figures and images for: The segregation of different submicroscopic imbalances underlying the clinical variability associated with a familial karyotypically balanced translocation
Source: Mol Cytogenet. 2015 Dec 30;8:106. doi: 10.1186/s13039-015-0205-9 (PMC4696321; doi:10.1186/s13039-015-0205-9)

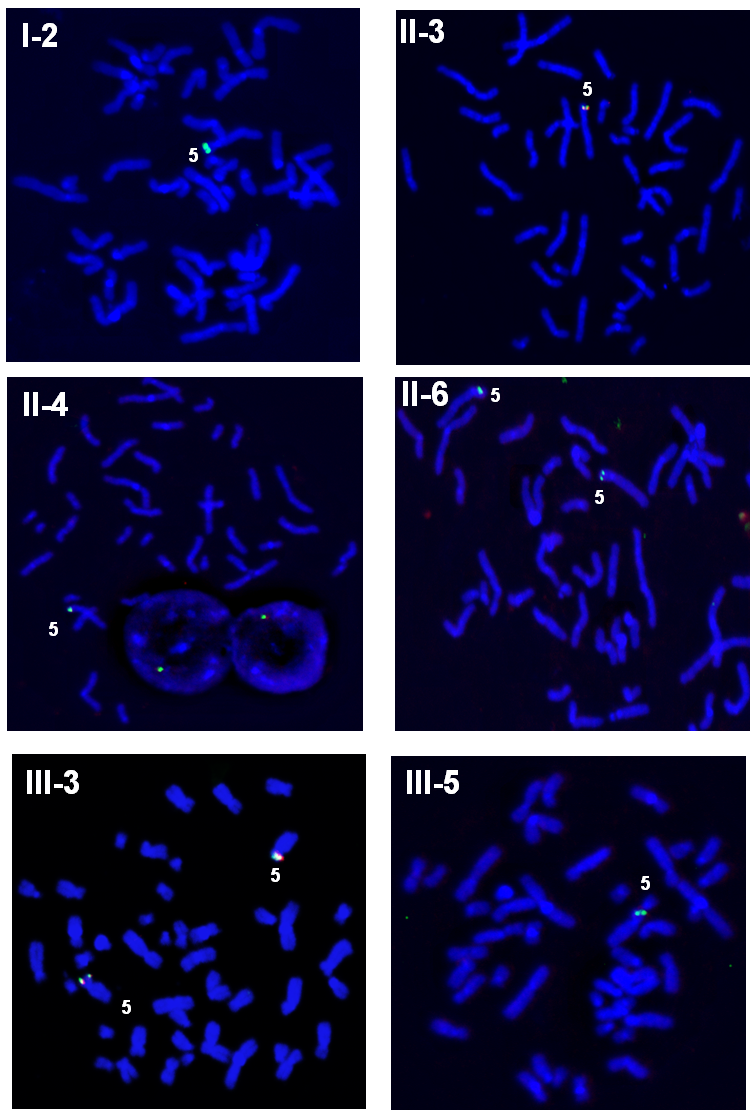

Supplement: Additional file 4: Figure S1. — Segregation of the 5p15.1 deletion. FISH using probes RP1-137K24 (red signal) and RP1-167G20 (green signal), mapping to the deleted segment at 5p15.1, revealed signals only on the normal chromosome 5 on metaphase spreads of the mother (II-4), maternal grandmother (I-2), aunt (II-3), and in the phenotypically normal brother (III-5) of the proband. Both probes hybridized to both chromosomes 5 on metaphases of the affected brother (III-3) and uncle of the proband (II-6). These results demonstrated that the deletion at 5p15.1 did not segregate with the clinical phenotype in the family. (TIFF 2444 kb) [file 13039_2015_205_MOESM4_ESM.tiff]

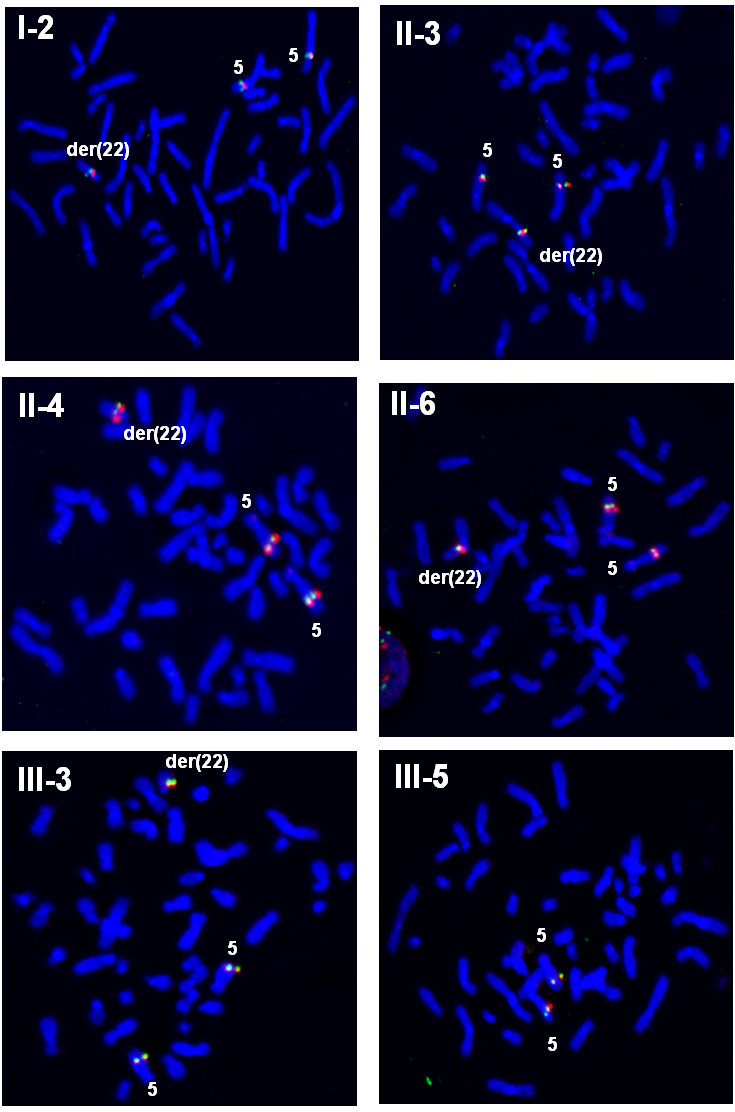

Supplement: Additional file 5: Figure S2. — Segregation of the 5q23.2-q23.3 duplication. FISH using probes RP11-48C14 (red signal) and RP1-236L2 (green signal), mapping to the duplicated segment, revealed signals on both chromosomes and an addition signal on the derivative chromosome 22, on metaphases spreads of all affected individuals in the family. Hybridization signals were only observed on both chromosomes 5 in the proband’s normal brother (III-5). These results demonstrated that the duplication at 5q23.2-q23.3 segregated with the clinical phenotype in the family. (TIFF 2394 kb) [file 13039_2015_205_MOESM5_ESM.tiff]

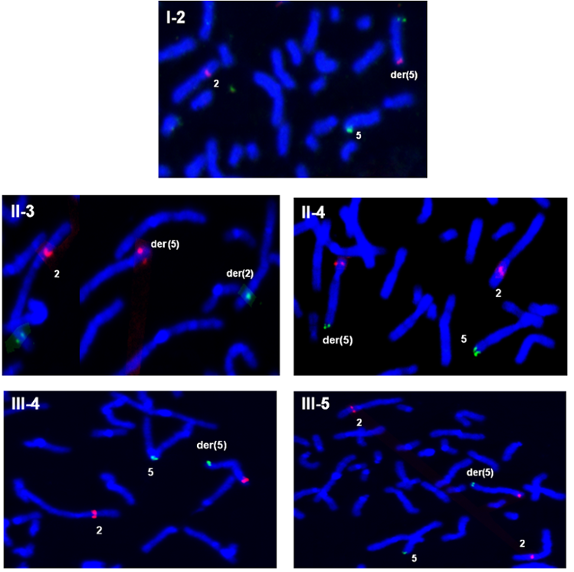

Supplement: Additional file 6: Figure S3. — Segregation of the 2p14 insertion into the short arm of chromosome 5. FISH using probe RP11-263L17 (red signal), mapping at 2p14, hybridized to the normal chromosome 2 and to the derivative 5 on metaphase spreads of the proband (III-4), his grandmother (I-2), mother (II-4) and aunt (II-3). On metaphase spreads of the phenotypically normal brother of the proband (III-5), probe RP11-263 L17 hybridized to the normal chromosome 2 and to the derivative 5. Probe RP11-281O15, mapping at 5q35.3 (green signals), was used as control for patients I-2, II-4, III-4 and III-5. Probe RP11-325M10 mapping at 2q33.3 (green signal), was used as control for patient II-3. These results indicated that the 2p14 segment was inserted into the chromosome 5 short arm, the same chromosome harbouring the 5p15.1 deletion. The phenotypically normal brother of the proband (III-5), being a carrier of the der(5), had a 2p14 duplication. The proband’s affected brother (III-3) and uncle (II-6), who did not carry the der(5), had the corresponding deletion (Fig. 3). (TIFF 962 kb) [file 13039_2015_205_MOESM6_ESM.tiff]

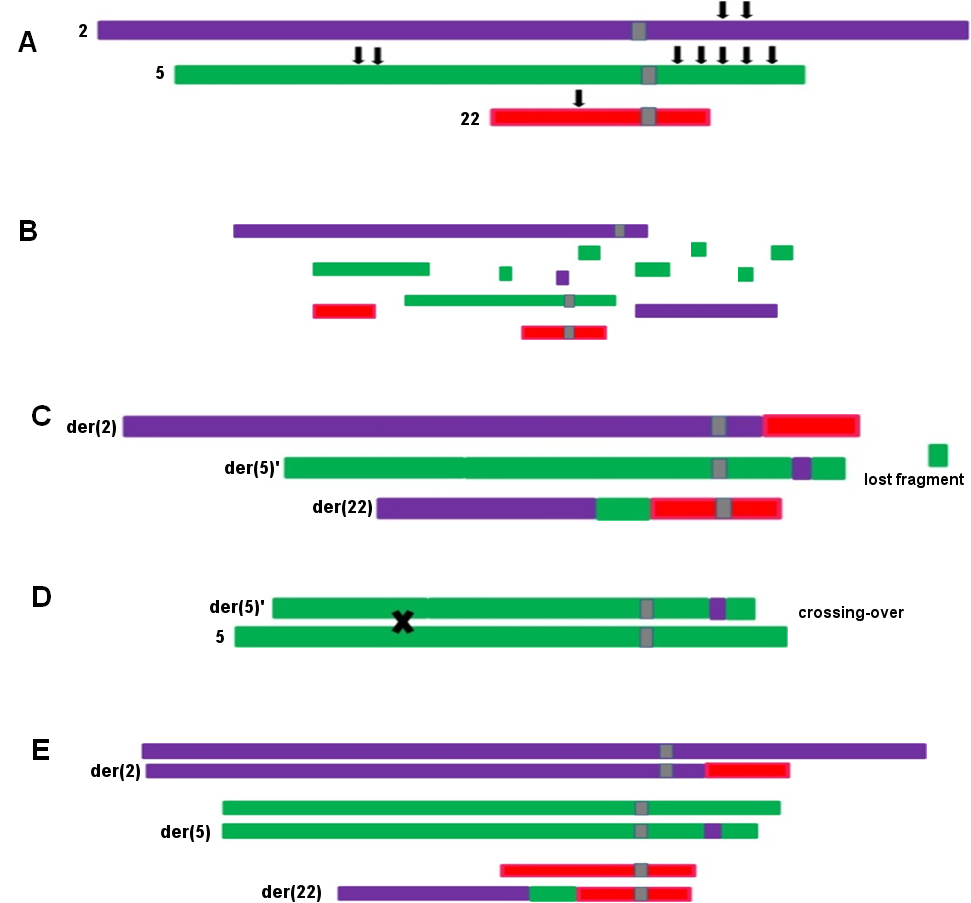

Supplement: Additional file 8: Figure S4. — Mechanism of formation of the t(2;5;22) based on MPS data. (A) The rearrangement might have originated from ten occurring breaks (arrows): two on the short arm of chromosome 2, five breaks on the short arm and two on the long arm of chromosome 5, and one break on the long arm of chromosome 22. (B) The resulting fragments (C) rejoined randomly, a fragment from the short arm of chromosome 5 being lost. (D) This rearrangement presumptively originated in an ancestor of the proband’s grandmother, since she did not carry the der(5) with a deletion of the segment corresponding to the 5q23.2-23.3 fragment inserted into der(22). A meiotic exchange between the normal chromosome 5 and the original der(5) would give rise to the observed der(5) with a normal long arm and an insertion of the segment 2p14 into the short arm. (E) The derivative chromosomes 2, 5 and 22 detected in the family, and their normal homologues are depicted. (TIFF 3425 kb) [file 13039_2015_205_MOESM8_ESM.tiff]

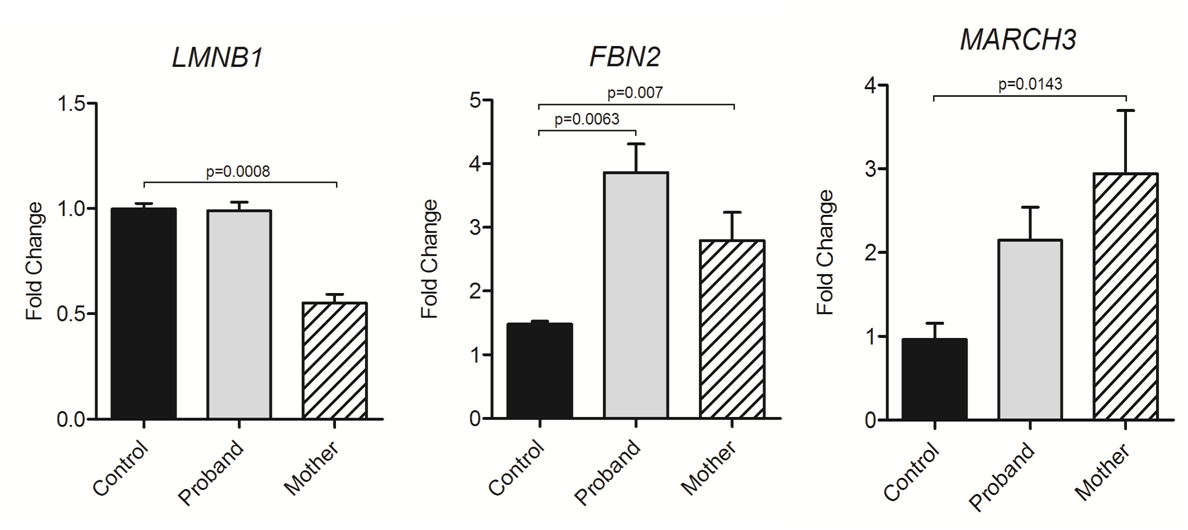

Supplement: Additional file 9: Figure S5. — LMNB1, MARCH3 and FBN2 expression determined by qRT-PCR. The diagram depicts the expression of LMNB1, MARCH3 and FBN2 in peripheral blood cells from the proband (III-4) and his mother (II-4), relative to healthy controls (n = 2). LMNB1 transcript levels did not differ between the proband and controls, but were decreased in his mother, both carriers of the duplication at 5q23.2-23.3, encompassing the LMNB1. The decreased levels of LMNB1 transcripts in the mother of the proband relative to her son might be due to an age-related effect [12]. On the other hand, increased transcript levels of FBN2 and MARCH3, also mapping to the duplicated interval, were observed in both carriers. All samples were tested in triplicate (diagram represents mean values), and the ACTB expression was used to calculate the relative and normalized levels of LMNB1, MARCH3 and FBN2 transcripts. Error bars denote standard error of the mean (SEM). P values for fold change in expression are indicated (unpaired Student's t test). (TIFF 1845 kb) [file 13039_2015_205_MOESM9_ESM.tiff]
